# *Apoxyria hirtuosa* (Wiedemann, 1821) comb. n., lectotype designation, redescription and identification key to species of *Apoxyria* Schiner, 1866  (Asilidae, Laphriinae)

**DOI:** 10.3897/zookeys.125.1790

**Published:** 2011-08-26

**Authors:** Edgar Alvim, Rosaly Ale-Rocha, Freddy Bravo

**Affiliations:** 1 Instituto Nacional de Pesquisas da Amazônia (INPA), Coordenação de Pesquisas em Entomologia, Av. André Araújo, 2936, Petrópolis, CEP 69011-970, Manaus, Amazonas, Brasil; 2 Fellowship CNPq; 3 Departamento de Ciências Biológicas, Universidade Estadual de Feira de Santana, Departamento de Ciências Biológicas, Av. Transnordestina S/N, Bairro Novo Horizonte 44036-900, Feira de Santana, BA, Brasil

**Keywords:** Neotropical, taxonomy, Laphystiini

## Abstract

The type specimens of *Neodiogmites hirtuosus* (Wiedemann, 1821), two males and one female, deposited at the Museum für Naturkunde der Humboldt-Universität (ZMHB), Berlin were examined. The specimens show the diagnostic characters of *Apoxyria* Schiner, 1866: face strongly pronounced, swollen and curved hind tibia, and terminalia with epandrium large and hypandrium short and obtuse. A new combination is suggested, *Apoxyria hirtuosa* (Wiedemann, 1821), and lectotype and paralectotypes are designated. The species is redescribed, the male terminalia is described and illustrated for the first time, and an identification key to *Apoxyria* is presented.

## Introduction

*Dasypogon hirtuosus* Wiedemann, 1821, a species classified in the subfamily Dasypogoninae (Papavero 2009), is known from Brazil, with no additional details of the collection locality, was described based on two males and one female without a holotype designation (i.e., syntypes) (Wiedemann 1821).Carrera (1949) transferred *Dasypogon hirtuosus* to *Laustauroides* Carrera, 1949 (Dasypogoninae) based on specimens collected in Brazil, however without examination of the type material. Subsequently, Artigas and Papavero (1988) synonymized *Laustauroides* with *Neodiogmites* Carrera, 1949, another genus of Dasypogoninae, and therefore the current name of the species is *Neodiogmites hirtuosus*.

During a revision of the genus *Neodigmites*, the three syntypes of *Neodigmites hirtuosus* were examined. It was apparent that this species is not a Dasypogoninae and consequently, is not a member of *Neodiogmites*. Here, we propose a new combination for this species, with a lectotype designation.

## Material and methods

The specimens studied are deposited in the Museum für Naturkunde der Humboldt-Universität (ZMHB), Berlin, Germany. The material includes three syntypes, two males and one female; and their labels read only “Brazil”. To observe the terminalia, it was detached from the abdomen and cleared in cold 10% KOH, followed by neutralization in acetic acid, dehydration in ethanol, and washing in distilled water. The dissected terminalia was placed in glycerin in a microvial pinned with the respective specimen. Morphological terms follow Cumming and Wood (2009).

## Taxonomy

### Apoxyria hirtuosa

(Wiedemann, 1821) comb. n.

http://species-id.net/wiki/Apoxyria_hirtuosa

Figs 1 –7

Dasypogon hirtuosus Wiedemann 1821: 227; Wiedemann 1828: 402 ( redescription); Walker 1854: 443 (check list); Schiner 1866: 679 (check list); Williston 1891: 67 (catalogue); Kertész 1909: 128 (catalogue);
Lastauroides hirtuosus ;Carrera 1949: 97, Fig. 27; 1958a: 146; Carrera and Vulcano 1961: 69 (prey); Carrera and Papavero 1962: 53 (check list); Hull 1962: 241, Figs 545, 1074, 1083 (check list); Martin and Papavero 1970: 29 (catalogue).
Neodiogmites hirtuosus ;Artigas and Papavero 1988: 213 (key), 151, Fig. 157; Papavero 2009:1 (catalogue);Geller-Grimm 2011 (online catalogue).

#### Type-material examined.

Lectotype male (ZMHB), present designation, labeled: “Brazil\ ?, [without date] V. Olfers coll.” A red label written “Lectotype” was added. Specimen in reasonable condition, head slightly dusty, flagellum and middle leg lost, right wing mounted on permanent slides, abdomen dissected and placed in a micro-vial with glycerin, pinned together with the specimen. Paralectotypes: 1 male and 1 female, same locality as lectotype. Paralectotype male (ZMHB ) in reasonable condition, head with a little dust, lacking flagellum, mesonotum broken posteriorly, abdomen cracked between the second and third segment. Paralectotype female (ZMHB ) in good condition, but the mesonotum is perforated posteriorly and the left flagellum is lost.

Lectotype male: Measurements: 12.5 mm (body length excluding antennae); 9.0 mm (wings).

#### Diagnosis.

Face pronounced, covered by yellow pruinescence; dorsocentral setae of the same length as the scutum setae, however black; scutellum covered by short yellow setae, with several yellow long and slender apical scutellar setae; wings with r1 open.

#### Redescription:

 **Lectotype male.** Head (Fig. 3): face black, covered by yellow pruinescence that is denser on the sides, pronounced, not ending abruptly on upper part, occupying 2/3 of face; mystax black and yellow with some yellow setae between the antenna and facial swelling; frons black with sparse yellow pruinescence, yellow setae below and beside ocellar tubercle; vertex black; ocellar tubercle with several yellow setae; orbital setae yellow;postocular setae black with thin yellow setae between them; occiput black with yellow pruinescence, with black setae and yellow lower setae; proboscis black, apex obtuse, with short yellow setae ventrally; palpus black, longer than half length of proboscis, yellow setae basally and black on the remainder; antenna black, scape and pedicel almost the same length with black setae, scape with some basal yellow setae.

**Figures 1–4.**
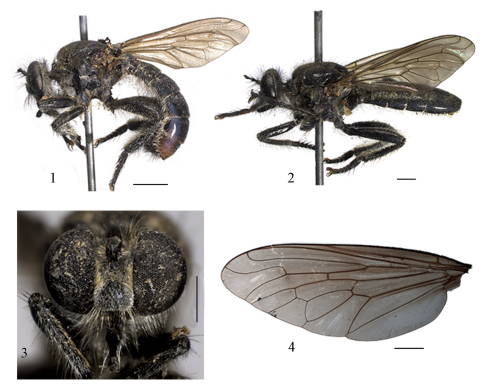
*Apoxyria hirtuosa* (Wiedemann, 1821) comb. n. **1** General lateral view of lectotype, male **2** General lateral view of paralectotype female **3** Frontal view of head of lectotype **4** Wing of lectotype. Scale = 1mm.

Thorax (Fig 1): black; second cervical sclerite black with sparse yellow pruinescence and black setae; antepronotum with black setae; postpronotum covered by yellow pruinescence and yellow setae laterally, some black setae mixed; postpronotal lobe with black setae anteriorly and yellow posteriorly; proepisternum and proepimeron with yellow pruinescence and yellow setae; scutum covered by short yellow setae; dorsocentral setae of the same length as the scutum setae, however black; two notopleural setae; three to four supra-alar setae and two postalar setae, all black; scutellum covered by short yellow setae, with several yellow long and slender apical scutellar setae; mesopleura covered by yellow pruinescence, except anteriorly on the anepisternum and katepisternum; anepisternum with yellow setae and some black setae posteriorly; katepisternum with yellow setae; katatergite with brownish setae.

Legs (Fig. 1): shiny black; coxae with yellowish pruinescence and yellow setae; femora covered by short yellow setae with some black setae on the dorsum; fore and hind tibiae covered by yellow setae, longer on ventral and posteroventral margin, and with dense short yellow setae on ventral margin which extend onto first tarsomere, these setae are also present on the hind tibiae, but are denser than on the other legs; fore tibia with anterodorsal, posterodorsal, and posteroventral rows of stout black setae; mid tibia with dorsal, posterodorsal, posteroventral, and ventral rows of stout black setae; hind tibia covered by yellow and black setae of different lengths, with anterodorsal, posterodorsal, and posteroventral rows of stout black setae and dense yellow setae on ventral and posteroventral margins. Tarsi covered by short yellow setae and stout black setae; claws black; pulvilli yellowish.

Wing (Fig. 4): membrane slightly infuscate; veins brown; alula reduced, but a small lobe is still distinct; r1 open, apex of R2+3 arching sharply anteriorly in 90° angle , R4 strongly sinuate, R4 and R5 diverge from each other at the wing margin, r5 open, m3 closed and petiolate; cell cup closed; haltere yellow.

**Figures 5–7.**
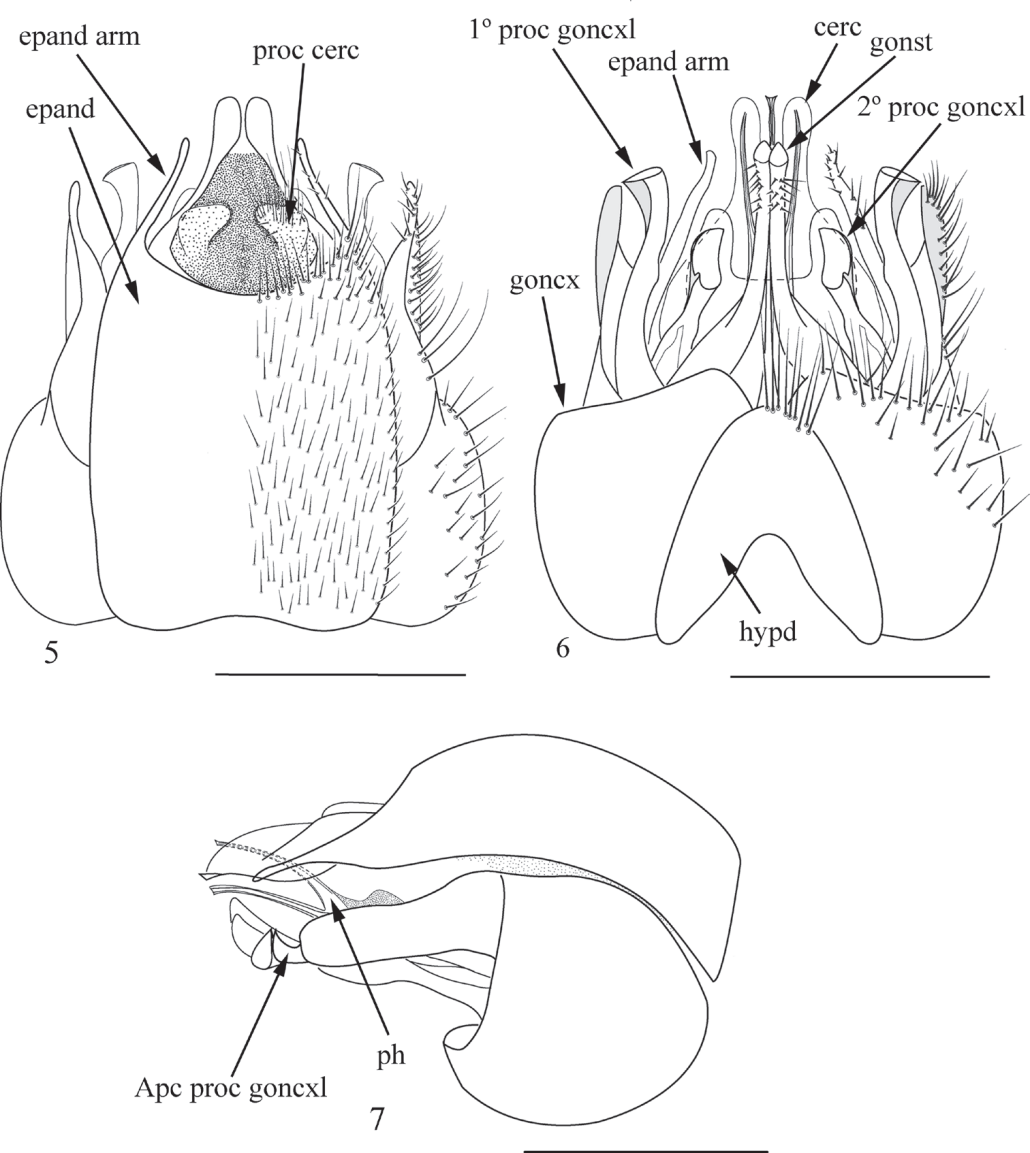
Terminalia of *Apoxyria hirtuosa* (Wiedemann, 1821) comb. n., lectotype male. **5** Dorsal **6** Ventral **7** Lateral. Scale = 1mm. Abbeviations: apc proc goncxl-apical process gonocoxal; cerc- cercus; epand- epandrium; epand arm- epandrial arm; 1° proc goncxl-first gonocoxal process; goncx-gonocoxito; gonst-gonostylus; hypd-hypandrium; proc cerc- process cercal; ph-phallus; 2° proc goncxl- second process gonocoxal.

Abdomen (Fig 1): black, covered by short yellow setae, longer on the sides of anterior three tergites; posterior margin of tergites 6 and 7 reddish. Terminalia (Figs 5–7): terminalia reddish with black setae; epandrium long, with deep, rounded sulcus on apical fourth, forming long arms laterally; subepandrial sclerite with medial evagination forming laterally arms with rounded apex; cercus with two projections dorsally; gonocoxite with a claw-shaped projection on the apex and with two expansions, the second expansion with shell-shaped apex; gonostylus with rounded apex; hypandrium short and boomerang-shaped.

Female (Fig. 2): flagellum longer than scape and pedicel combined, with and a spine on the apex, black covered by brown pruinescence denser in the base; postpronotal lobe with black setae anteriorly; scutellum with some short black setae anteriorly; legs and abdomen with fewer setae than in male; terminalia pale brown.

#### Distribution:

 Brazil.

#### Discussion.

After the original description of *Dasypogon hirtuosus* by Wiedemann (1821), the type specimens have not been examined until now. All the transfers to other genera were based on other specimens collected in Brazil (Carrera 1949; Artigas and Papavero 1988). The morphological study of the syntypes of this species revealed the lack of a spine on the prothoracic tibia, one of the most important characters to identify members of Dasypogoninae (Hull 1962; Papavero 1973; Dikow 2009). Moreover, the specimens studied have important characters that places them in the Laphriinae: the male with only six abdominal tergites visible dorsally, as defined by Hull (1962), wings whit r1 open, apex of R2+3 arching sharply anteriorly in 90° angle and R4 strongly sinuate (Dikow 2009).

Among the genera of Laphriinae, the specimens show the diagnostic characters of *Apoxyria*: numerous long and slender apical scutellar setae, the face strongly pronounced, swollen hind femora, swollen and curved hind tibia, and terminalia with a large epandrium and short and obtuse hypandrium.

At the moment, the genus *Apoxyria* is known only from Brazil, now with three species: *Apoxyria apicata* Schiner, 1866*, Apoxyria americana* Carrera, 1955 and *Apoxyria hirtuosa* .There are insufficient data to determine their distributions in detail, because few specimens of this genus have been collected.

## Key to species of *Apoxyria* Schiner, 1866.

**Table:** 

1	R2+3 ends in R1 (not reaching C), cell r1close, petiolate; male with posterior margins of tergites 6–7 black (Brazil)	*Apoxyria apicata* Schiner, 1866
–	R2+3 ends in C, cell r1 open (Fig. 4); male with posterior margins of tergites 6–7 either reddish or yellow	2
2	Mesonotum covered by short yellow setae (Fig. 1); male with posterior margins of tergites 6–7 reddish; epandrial arms long and slender (Fig. 5) (Brazil)	*Apoxyria hirtuosa* (Wiedemann, 1821) comb. n.
–	Mesonotum covered by short black setae; male with posterior margins of tergites 6–7 yellow; epandrial arms short and thick (Fig. 66 from Artigas et al. 1997) (Brazil: Goiás and Santa Catarina)	*Apoxyria americana* Carrera, 1955

## Supplementary Material

XML Treatment for Apoxyria hirtuosa
